# Newborn skin reflection: Proof of concept for a new approach for predicting gestational age at birth. A cross-sectional study

**DOI:** 10.1371/journal.pone.0184734

**Published:** 2017-09-20

**Authors:** Zilma Silveira Nogueira Reis, Gabriela Luiza Nogueira Vitral, Ingrid Michelle Fonseca de Souza, Maria Albertina Santiago Rego, Rodney Nascimento Guimaraes

**Affiliations:** 1 Center of Health Informatics, Faculty of Medicine, Universidade Federal de Minas Gerais, Belo Horizonte, Minas Gerais, Brazil; 2 Postgraduation Program of Women’s Health, Faculty of Medicine, Universidade Federal de Minas Gerais, Belo Horizonte, Minas Gerais, Brazil; 3 Department of Gynecology and Obstetrics, Faculty of Medicine, Universidade Federal de Minas Gerais, Belo Horizonte, Minas Gerais, Brazil; 4 Department of Pediatrics, Faculty of Medicine, Universidade Federal de Minas Gerais, Belo Horizonte, Minas Gerais, Brazil; Centre Hospitalier Universitaire Vaudois, FRANCE

## Abstract

**Background:**

Current methods to assess the gestational age during prenatal care or at birth are a global challenge. Disadvantages, such as low accessibility, high costs, and imprecision of clinical tests and ultrasonography measurements, may compromise health decisions at birth, based on the gestational age. Newborns’ organs and tissues can indirectly indicate their physical maturity, and we hypothesized that evolutionary changes in their skin, detected using an optoelectronic device meter, may aid in estimating the gestational age. This study analyzed the feasibility of using newborn skin reflectance to estimate the gestational age at birth noninvasively.

**Methods and findings:**

A cross-sectional study evaluated the skin reflectance of selected infants, preferably premature, at birth. The first-trimester ultrasound was the reference for gestational age. A prototype of a new noninvasive optoelectronic device measured the backscattering of light from the skin, using a light emitting diode at wavelengths of 470 nm, 575 nm, and 630 nm. Univariate and multivariate regression analysis models were employed to predict gestational age, combining skin reflectance with clinical variables for gestational age estimation. The gestational age at birth of 115 newborns from 24.1 to 41.8 weeks of gestation correlated with the light at 630 nm wavelength reflectance 3.3 mm/6.5 mm ratio distant of the sensor, at the forearm and sole (Pearson’s correlation = 0.505, P < 0.001 and 0.710, P < 0.001, respectively). The best-combined variables to predict the gold standard gestational age at birth was the skin reflectance at wavelengths of 630 nm and 470 nm in combination with birth weight, phototherapy, and adjusted to include incubator stay, and sex (R^2^ = 0.828, P < 0.001). The main limitation of the study is that it was very specific to the premature population we studied and needs to be studied in a broader spectrum of newborns.

**Conclusions:**

A novel automated skin reflectometer device, in combination with clinical variables, was able to predict the gestational age and could be useful when the information is in doubt or is unknown. Multivariable predictive models associated the skin reflectance with easy to obtain clinical parameters, at the birth scenario. External validation needs to be proven in an actual population with the real incidence of premature infants.

## Introduction

Timely decisions about the immediate care at birth often depend on the gestational age (GA). Perinatal morbidity and mortality are frequently associated with short gestation at birth and with low birth weight in pregnancy [1]. Premature newborns are more likely to die during the first hours of life or to develop lifelong complications [2]. These infants need critical attention to survive, and their age is one of the primary predictors of neonatal outcomes [3]. The current methods to assess the GA during prenatal care or at birth have disadvantages, such as low accessibility, high costs, and imprecision of results [4–6].

Theoretically, fetal age begins at conception, but this information is difficult to determine accurately. Unknown or inaccurate last menstrual period (LMP) dates result in misclassification of newborns at birth, impacting the proportions of preterm and post-term groups, and resulting in inexact proportions of small for gestational age infants and large for gestational age infants [5, 7, 8]. The gold standard for determining GA is the early obstetric ultrasound assessment that establishes or confirms the number of weeks of gestation during the first trimester [4].

While the GA estimation by current approaches faces challenges, fetal maturity may be indirectly determined based on their organs and tissues. Evolutionary changes in the skin of neonates contribute to the maturity scores at birth, together with other external and neuromuscular indicators [9, 10]. The connection of an age-related morphologic pattern of the fetal skin allowed determination of fetal age in human postmortem examination, with high concordance with LMP and early ultrasound [11]. The skin development during intrauterine life is a continuum that involves the juxtaposition and interaction of mesodermal and ectodermal tissues to form a protective barrier, as expected in term neonates [12]. The period of 22 to 40 weeks of gestation correlates with the maturation of the stratum corneum, the biggest protection against dehydration, heat loss, and injury, and is essential for the health of the preterm infant [13].

The corneum stratum, as well other superficial layers of the skin, can be penetrated by light through its thickness and all its components. Accordingly, the surface structure and tissue composition can be noninvasively accessed by light [14]. Optoelectronic systems can obtain backscattered light signals, captured on a photo-detector, and estimate skin fat thickness measurements [15]. Certain wavelengths of the electromagnetic spectrum have the potential to contribute to the prediction of skin thickness and other skin properties. The skin reflectance values, obtained using spectrophotometry, were different between black and white skin, and female and male newborns [16]. Reflectance at 837 nm increased exponentially with the gestational age, independent of race or sex [17].

Based on the rationale of the skin development during fetal life, this study tested the feasibility of using the neonatal skin reflectance to noninvasively estimate the GA at birth with an optoelectronic device, combined with clinical data, in a multivariable predictive model. We hypothesized that light scattering by the skin mainly occurs because of the presence of keratin and collagen in the superficial layers of the skin, which is still developing during the gestational period.

## Methods and materials

### Environment and subjects

This cross-sectional study evaluated a selection of infants that were born from August 2016 to June 2017, in two Brazilian maternity hospitals, according to inclusion and exclusion criteria. The protocol of research was approved by the institutional review boards in Brazil, the Ethics Committees of the Universidade Federal de Minas Gerais, with a national register number in Plataforma Brasil: CAAE 49798915.2.0000.5149. A written informed consent was obtained from each mother on behalf the newborns. The actual birth and neonatal care took place in the university hospital of Universidade Federal de Minas Gerais and the Hospital Sofia Feldman, a public health institution. Both are referral settings for high-risk pregnancies in the perinatal network of the city, Belo Horizonte, Brazil.

The eligibility criteria for subject selection were alive infants who were born with gestational age above 25 weeks of gestation with gestational age calculated using early ultrasound, done before 14 weeks, which is the gold standard for aging based on ultrasound [4]. Fetal diseases that can affect the skin structure, such as fetal hydrops, genodermatoses, absolute oligohydramnios, as observed in Potter sequence, or clinical evidence of intrauterine infections, were not included. In order to obtain a continuum of the skin response along the GA, premature infants were the primary targets during the selection, even if the sample does not represent the real incidence of birth in each week of gestation. The study size was planned based on a previous moderate correlation between the gestational age and the skin reflection [17].

The newborn assessment occurred twice during the first 48 hours after delivery. The first one occurred as soon as possible after birth and the second one was approximately 24 hours from the first. The skin reflectance was measured in two places on each infant’s body, over the anterior distal forearm, and on the sole of the foot. This choice attended the patient security recommendation for minimum manipulation of very-low-birth newborns in NICU. The live newborns were evaluated in the nursing care unit, either on the mother’s lap or inside an incubator. In the neonatal intensive care unit (NICU), the assessment occurred inside their incubators or in an open heating crib, when that is where they were being cared for, in order to ensure minimum handling and stable clinical conditions. The phototherapy, when in progress, was turned off during the assessment. Obstetric and clinical variables of the neonates were recorded. The Fenton growth chart for preterm infants was the standard for nutritional classification at birth, based on the birth weight [18].

### The optical prototype and the experimental protocol

**Fig 1:**
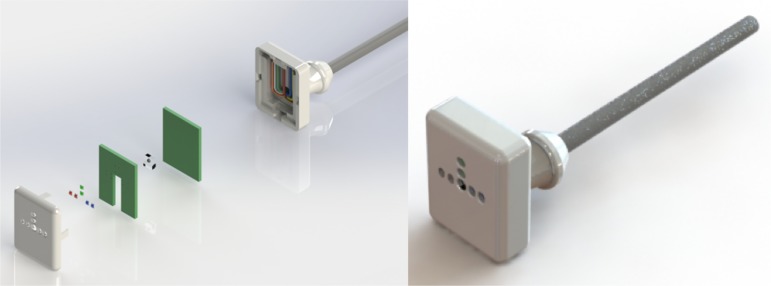
Prototype of the sensor probe. (A) Exploded perspective drawing of optoelectronic components fitting in the cover probe. (B) 3D illustration of the second version of the sensor module. Note: Exact positions between the LEDs and photodiode, in the middle of the sensor are: Red 1 = 3.3 mm; Red 2 = 6.5 mm; Green 1 = 4.0 mm; Green 2 = 7.2 mm; Blue 1 = 3.3 mm; Blue 2 = 6.5 mm.

A noninvasive, handheld, and low cost optoelectronic device was developed to measure the backscattered light signal from the skin, according to the skin thickness and the composition of the tissue. University bioengineering, physics and electronic technician in the Health Informatics Center constructed the prototype. The expectation was that the backscattered light could exhibit modifications according to the skin growing, mirroring the gestational evolution. The prototype is composed of a sensor probe, unit controller, and power supply. The sensor probe is comprised of two blue, two green, and two red light emitting diodes (LEDs) at wavelengths of 470 nm, 575 nm, and 630 nm, a photodiode, a printed circuit board, and an optical barrier that surrounds the photodiode (Fig 1). A sensor converts the light skin response into a frequency that is directly proportional to the amount of reflected light. The LEDs of the same color were positioned side by side at different distances from the photodiode, resulting in six frequency acquisitions: red, green, and blue, positioned at approximately 3.3 mm and 6.5 mm from the emitter. The unit controller recorded eight automated values: the ambient light, the dark current, and one measurement from each LED. The data acquisitions occurred automatically, when the sensor touched the skin, without operator influence, and recorded one time per newborn and location on the body. A cover for the probe, which was easily disinfected, was developed, using a three dimensional printer, and proper user ergonomics, to avoid intense pressure from the operator against the newborn's skin, to keep the reflectivity measurements stable. Details of the sensor design, the signal processor, and the following process of gestational age estimation were patented under number BR1020160256020 on behalf of the Universidade Federal de Minas Gerais, Brazil and their inventors [19].

### Data processing and statistical analyses

The descriptive statistics explored the demographic and clinical characteristics of the infants, using frequency, measures of central tendency, and variability, according to the groups of interest, premature and term infants. To evaluate intraobserver variability, additional 122 measurements were obtained in 61 adults. The skin over hand was evaluated, twice and sequentially, by the same observer, with the observer blinded to the previous measurement. One hundred twenty-two measurements were obtained in the same group of 61 adults to assess interobserver variability. The skin over hand was evaluated by a second blinded examiner, with a single measurement being acquired by each one. Intraobserver and interobserver repeatability were assessed using the intraclass correlation coefficient (ICC). Bland–Altman plots [20] were obtained to evaluate the systematic bias between the two measurements for skin reflectance at 470 nm, 575 nm and 630 nm acquisitions, distant near and far from the sensor.

Inferential statistical analysis evaluated the correlation between the skin reflectance and the GA. The sensor acquisitions produced by the skin reflection at 470 nm (R_470_), 575 nm (R_575_), and 630 nm (R_630_), at 3.3 mm and 6.5 mm of distance of the sensor, and their ratios within the same color, were the independent variables. Influence of sex, phototherapy, and incubator environment on the skin reflection ratio was analyzed using a Student t-Test. Paired samples Student-test compared repeated measurements between the first and second day after birth. The best body site on the newborn skin was inferred from the regression coefficients obtained in the graph frequency versus GA. Univariate and multivariate models of regression analysis were employed to estimate the correlation of predictors with the GA. Pearson correlation was the first step, but other non-linear models were adjusted to fit the correlation between predictors and outcome better. Multiple regression analysis included predictor variables from the univariate models, considering the effect modifiers from sex, birth weight, incubator staying, and phototherapy (input and output, P-value of 0.10), using the backward method of model arrangement. The fit of the models and calibration, specifically the ANOVA and Durbin-Watson test for residuals, were performed. Coefficients of determination (adjusted R^2^) were carried out based on the hypothesis that all coefficients were 0. The statistical program SPSS^®^ 22.0 was used for the analysis. The significance levels, adjusted for the hypothesis test, were 5% and 95% (Confidence Interval (95% CI)).

## Results

**Fig 2:**
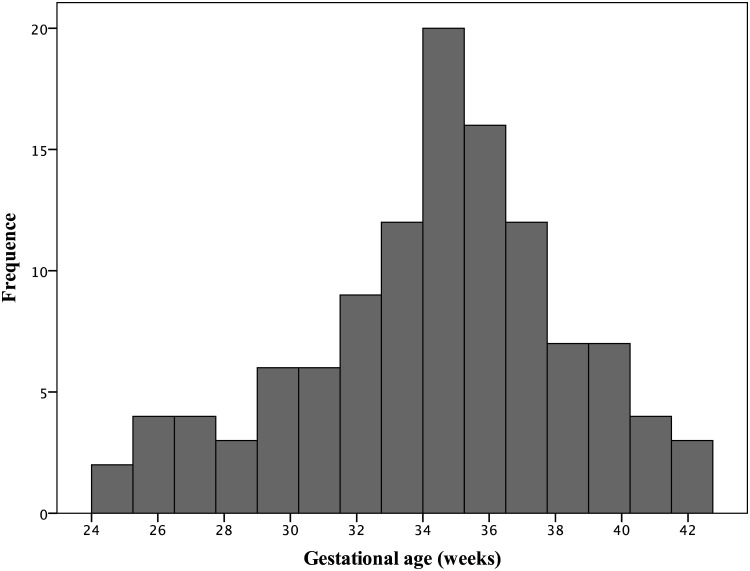
Gestational age distribution in the selected sample of newborns. This was calculated by using the gold standard approach [4]. Normal distribution (Kolmogorov-Smirnov > 5%).

One, out of the 117 examined newborns, was excluded because the parents withdrew the voluntary authorization for participation. The other exclusion was an outlier for all measurements considered without recuperation. The dependent variable, GA at birth, ranged from 24.1 to 41.8 weeks of gestation. The distribution of their values had a normal standard distribution, with a mean of 34.1 ± 4.1 weeks (95% CI, 33.3 to 34.8) (Fig 2). Most of the subjects had neonatal complications related to high-risk pregnancy and 78% of them were premature (Table 1).

**Table 1:** Clinical characteristics of the studied newborns (n = 115).

Clinical and obstetric characteristics	N	Descriptive statistics
Gestational age, average (SD), weeks	115	34.1 (4.1)^#^
Prematurity n (%)	115	90 (78.3)
Major malformations n (%)	115	9 (7.8)
Twinning n (%)	115	17 (14.8)
Male/female n (%)	115	58 (50.4)/57 (49.6)
NICU at the first assessment n (%)	115	66 (57.4)
Incubator staying n (%)	115	64 (55.7)
Phototherapy at the first assessment n (%)	115	12 (10.4)
Birth weight, average (SD), kg	113	2171.4 (783.8)^#^
Small for gestational age* n (%)	113	24 (21.2)
Large for gestational age* n (%)	113	3 (2.7)

SD: standard deviation; NICU: neonatal intensive unit care

^#^Normal distribution (Kolmogorov-Smirnov > 5%)

*Based on Fenton growth-chart [18]

### The newborn skin reflection acquisitions

Regarding the acquisitions, access to the skin reflection from the sole was possible in all of the newborns. In the first 24 hours of life, 113 (98.3%) of the neonates had the forearm region available, depending on which interventions, such as equipment, catheters, and medical devices, were being used for the NICU newborns. Most (76.5%, n = 88) of them had sensors and vascular access at the time of the examination. During the second day of life, 107 (96.8%) and 109 (94.9%) had both the sole and forearm reachable, respectively.

The skin reflection intensity of the skin over the forearm and from the sole, the backscattering dropped on the second day for the R_630_ at 6.5 mm, and for the R_575_ at 4.0 mm and R_470_ at 3.3 mm. Forearm skin reflections were different from the ones obtained at the sole position (Table 2).

**Table 2:** Skin reflection and location of assessment at the first and second day after birth, obtained with the optical prototype.

LED specifications (Reflectance values/10^6^)	Forearm Mean (SD)			Sole Mean (SD)			Forearm vs. Sole reflectance difference	
	Day 1 (n = 113)	Day 2 (n = 107)	Day 1 vs Day 2 P-value*	Day 1 (n = 115)	Day 2 (n = 109)	Day 1 vs Day 2 P-value*	Day 1 (n = 228) P-value**	Day 2 (n = 216) P-value**
R_630_ at 6.5 mm	0.085 (0.033)	0.078 (0.031)	**0.003**^#^	0.108 (0.030)	0.094 (0.028)	**< 0.001**^#^	**< 0.001**^#^	**0.001**^#^
R_630_ at 3.3 mm	0.444 (0.150)	0.434 (0.144)	0.312	0.507 (0.092)	0.497 (0.099)	0.286	**0.001**^#^	**0.001**^#^
R_575_ at 7.2 mm	0.001 (0.002)	0.001 (0.001)	0.108	0.002 (0.002)	0.001 (0.001)	0.071	0.256	0.095
R_575_ at 4.0 mm	0.004 (0.002)	0.003 (0.001)	**0.025**^#^	0.004 (0.003)	0.004 (0.002)	**< 0.001**^#^	**0.004**^#^	**0.025**^#^
R_470_ at 6.5 mm	0.027 (0.039)	0.025 (0.037)	0.734	0.026 (0.007)	0.032 (0.042)	0.104	0.833	0.167
R_470_ at 3.3 mm	0.202 (0.163)	0.249 (0.036)	**< 0.001**^#^	0.0247 (0.089)	0.023 (0.079)	**0.012**^#^	**0.011**^#^	**< 0.001**^#^

LED: Light emitting diode; R_630:_ skin reflectance at 630 nm; R_575_: skin reflectance at 575 nm; R_470:_ skin reflectance at 470 nm;

*P-value: Paired Student t-Test;

**P-value: Independent Student t-Test;

^#^Significant association.

### Repeatability of acquisitions

The ICC of differences between repeat acquisitions by the same observer was 0.964 (95% CI, 0.941–0.978) for R_630_ at 3.3 mm, 0.986 (95% CI, 0.977–0.988) for R_630_ at 6.5 mm, 0.996 (95% CI, 0.994–0.998) for R_575_ at 7.2 mm, 0.992 (95% CI, 0.987–0.995) for R_575_ at 4.0 mm, 0.982 (95% CI, 0.970–0.989) for R_470_ at 6.5 mm, 0.965 (95% CI, 0.943–0.982) for R_470_ at 3.3 mm. The interobserver ICC for repeatability was 0.919 (95% CI, 0.869–0.951) for R_630_ at 3.3 mm, 0.881 (95% CI, 0.810–0.927) for R_630_ at 6.5 mm, 0.858 (95% CI, 0.775–0.913) for R_575_ at 7.2 mm, 0.807 (95% CI, 0.698–0.880) for R_575_ at 4.0 mm, 0.922 (95% CI, 0.874–0.953) for R_470_ at 6.5 mm, 0.898 (95% CI, 0.836–0.938) for R_470_ at 3.3 mm. The relationship between mean skin reflectance values and both intra- and interobserver differences are shown in S1–S12 Figs.

### Gestational age prediction based on the newborn skin reflection

The skin reflectance in the red wavelength (R_630_) range was depending on GA. The univariate linear analysis showed a moderate correlation between skin reflection and GA in the skin over the sole and forearm, assessed during the first day of life, in the total group of newborns. The reflectance from LEDs at 3.3 mm/6.5 mm ratio was best associated with the weeks of gestation at the skin of the sole: r = 0.682, P < 0.001 and r = 0.710, P < 0.001, for the premature infants and the overall group of newborns, respectively (Table 3). The skin reflectance in the green 575 nm wavelength (R_575_) had no significant linear correlation with the GA (Table 3).

**Table 3:** Univariate analysis of the correlation between the skin reflection and gestational age at the first day after birth, obtained with the optical prototype.

Predictors	N	Premature infants Linear coefficient (P-value*) n = 90	Total newborns Linear coefficient (P-value*) n = 115
**Forearm**			
R_630_ at 6.5 mm	113	-0.489 (< 0.001)^#^	-0.006 (0.952)^#^
R_630_ at 3.3 mm	113	-0.0283 (0.795)	0.433 (< 0.001)^#^
R_630_ 3.3 mm/6.5 mm ratio	113	0.599 (< 0.001)^#^	0.505 (< 0.001)^#^
R_575_ at 7.2 mm	113	-0.148 (0.167)	-0.086 (0.365)
R_575_ at 4.0 mm	113	-0.123 (0.254)	-0.095 (0.3165)
R_575_ 4.0 mm/7.2 mm ratio	113	0.244 (0.022) ^#^	-0.071 (0.457)
R_470_ at 6.5 mm	113	0.041 (0.706)	0.076 (0.457)
R_470_ at 3.3 mm	113	0.110 (0.307)	0.239 (0.011)^#^
R_470_ 3.3 mm/6.5 mm ratio	113	0.223 (0.037) ^#^	0.256 (0.006)^#^
**Sole**			
R_630_ at 6.5 mm	115	-0.457 (< 0.001)^#^	-0.405 (<0.001)^#^
R_630_ at 3.3 mm	115	0.355 (0.001) ^#^	0.500 (< 0.001)^#^
R_630_ 3.3 mm/6.5 mm ratio	115	0.682 (< 0.001)^#^	0.710 (< 0.001)^#^
R_575_ at 7.2 mm	115	-0.260 (0.013) ^#^	-0.099 (0.294)
R_575_ at 4.0 mm	115	-0.178 (0.094)	-0.080 (0.396)
R_575_ 4.0 mm/7.2 mm ratio	115	0.265 (0.012)	0.017 (0.856)
R_470_ at 6.5 mm	115	0.027 (0.799)	0.233 (0.012) ^#^
R_470_ at 3.3 mm	115	0.402 (< 0.001)^#^	0.542 (< 0.001)^#^
R_470_ 3.3 mm/6.5 mm ratio	115	0.441 (< 0.001)^#^	0.435 (< 0.001)^#^

R_630_: skin reflectance at 630 nm; R_575_: skin reflectance at 575 nm; R_470_: skin reflectance at 470 nm;

*P-value: Pearson’s correlation;

^#^Significant correlation.

**Fig 3:**
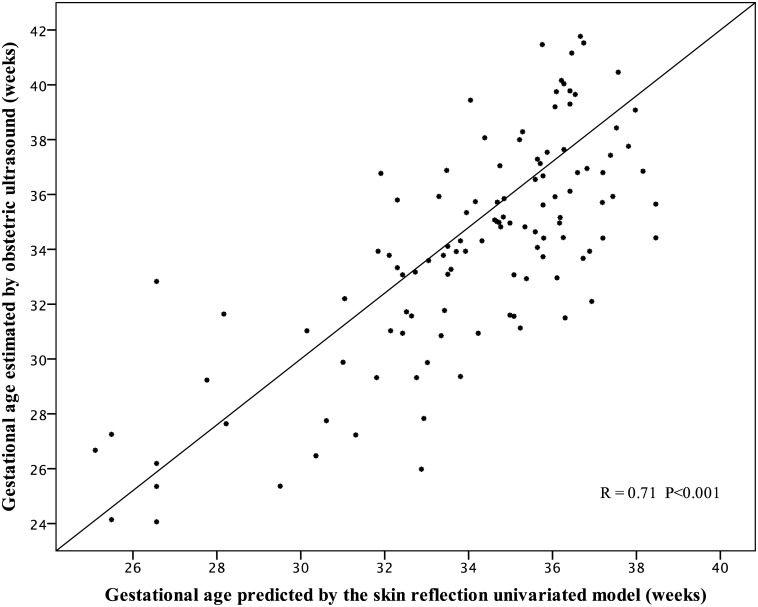
Gestational age estimated by the skin reflectance, during the first day of life, at the skin of the sole of foot vs. gestational age by early obstetric ultrasound.

**Fig 4:**
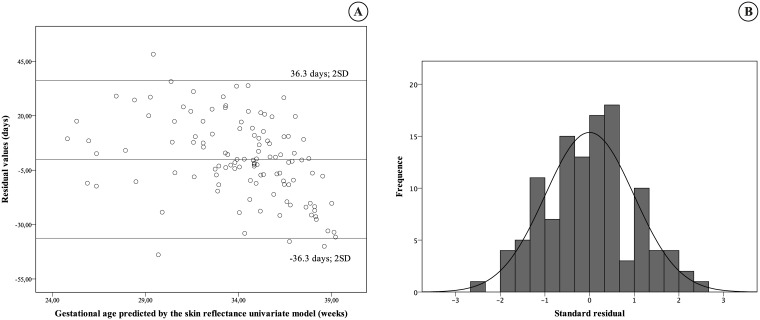
Standard deviation of residual values and histogram of residual value for the skin reflectance vs. gestational age. (A) Residual values (B) Histogram of residual values. This was during the first day of life, at the skin of the sole of the foot.

The best-adjusted univariate model to explain the correlation between the sole skin R_630_ reflection ratio (3.3 mm/6.5 mm ratio, considering distance of sensor) and the GA was an inverse function equation: $GA=44.75\;-\frac{48.93}{{\text{R}}_{630}\;\frac{3.3}{6.5}\text{mm}\;\text{ratio}}$ R^2^ = 0.56, P < 0.001. Predicted values versus GA estimated by obstetric ultrasound had moderate correlation, R = 0.71, P < 0.001 (Fig 3). The assessment occurred at the skin of the sole, during the first day of life. The residual values of GA that were not explained by the univariate model had a normal distribution with an estimated error of 18.5 days of gestation, based on the residual values. It means that 95% of occasions, GA calculated by the model differed until 36.3 days from GA estimated by obstetric ultrasound. The values were plotted with the mean and two SD in Fig 4.

### Performance of models to predict GA based on the newborn skin reflection, including clinical variables adjustments

The skin reflection at the sole skin, R_630_ 3.3 mm/6.5 mm ratio, considering distance of sensor, was influenced by clinical variables. For phototherapy, mean backscattering had values 4.16 ± 1.05 and 5.05 ± 1.18 for receiving phototherapy and no phototherapy, respectively (P = 0.023) and for incubator staying, mean backscattering was 5.58 ± 0.92 and 4.40 ± 1.13, P < 0.001 for staying inside the incubator or not, respectively. Regarding sex, mean backscattering was not different for male and female: 5.06 ± 1.21 and 4.73 ± 1.16, P = 0.136.

Compositions with the skin backscattering to R_470_ and R_630_, and ratios within the same colors, from the sole location on the newborn skin, were proposed as independent variables for the multivariable analysis. Table 4 introduces the final model parameters. In this selected group of neonates, 66.4% of the variability of GA was explained based on the skin reflectance.

**Table 4:** Predictor variables for gestational age at birth, based on the skin reflection at the sole of the newborn, adjusted for clinical variables (n = 113).

Predictors	Univariate Analysis Crude Correlation		Multiple Analysis Adjusted Correlation	
	Beta coefficient (95% CI)	R^2^ (P-value*)	Adjusted Beta Coefficient (95% CI) P-value	Adjusted R^2^ (P-value*)
**Model 1**				
1/R_630_ 3.3 mm/6.5 mm ratio	-48.9 (-56.9 to -40.9)	0.564 (< 0.001)	-44.4 (-53.1 to -35.8) P < 0.001	0.667 (< 0.001)
R_470_ at 3.3 mm^@^	24 (17 to 31)	0.293 (< 0.001)	15 (10 to 20) P< 0.001	
**Model 2**	Beta coefficient (95% CI)	R^2^ (P-value*)	Adjusted Beta Coefficient (95% CI) P-value	Adjusted R^2^ (P-value*)
1/R_630_ 3.3 mm/6.5 mm ratio	-48.9 (-56.9 to -40.9)	0.564 (< 0.001)	-20.9 (-27.7 to -14.4) P < 0.001	0.828 (< 0.001)
R_470_ at 3.3 mm^@^	24 (17 to 31)	0.293 (< 0.001)	6 (2 to 10) P = 0.007	
Birth weight (grams)	0.004 (0.003 to 0.004)	0.748 (< 0.001)	0.003 (0.002 to 0.003) P < 0.001	
Incubator^##^	-5.3 (-6.4 to -4.1)	0.430 (< 0.001)	-0.77 (-1.6 to 0.1) P = 0.089	
Phototherapy^##^	-4.9 (-6.2 to -1.6)	0.089 (0.001)	Excluded (0.478)	
Sex^#^	-0.8 (-2.3 to 0.7)	0.008 (0.182)	Excluded (0.552)	

^@^value*10^−6^;

^#^1 = male;

^##^1 = yes;

*Adjust of the model, ANOVA;

Constant for Model 1 = 39.5; Constant for Model 2 = 32.3.

**Fig 5:**
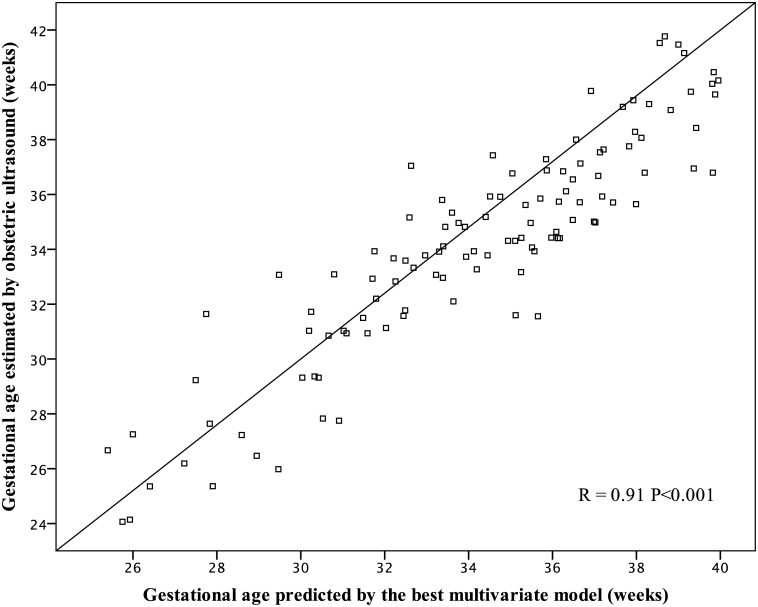
Title gestational age estimated by the multivariate model vs. gestational age by the early obstetric ultrasound.

**Fig 6:**
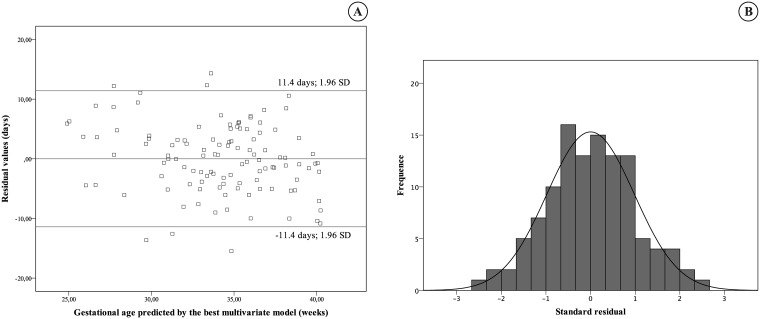
Standard deviation of residual values and histogram of residual value for the best multivariate model vs. gestational age. (A) Residual values (B) Histogram of residual values. This was during the first day of life, at the skin of the sole of the foot.

Modeling multiple linear regressions, models were based on combinations of skin backscattering and clinical data. The best model for gestational age prediction achieved R^2^ = 0.828, adjusting the R_630_ 3.3 mm/6.5 mm ratio and R_470_ at 3.3 mm to birth weight, incubator staying, phototherapy on the first day of life, and sex. Predicted values versus GA estimated by obstetric ultrasound had correlation R = 0.91, P < 0.001 (Fig 5). The residual analysis presented a normal distribution and estimated error of 5.8 days for the GA, based on the standard residual. It means that 95% of occasions, GA calculated by the model did not differ more than 11.4 days from GA estimated by obstetric ultrasound (Fig 6).

## Discussion

In this study, we used a new approach to estimate indirectly the GA at birth, based on the optical properties of the human skin and on knowledge about the maturity of the skin, during fetal life. To obtain data, the emitter and sensor probe developed just touched the skin, for a few seconds. An electronic platform prototype with an embedded software controller made the assessment very simple and the assessment was automated by the processor. The plan for development of this process showed that this is an affordable and noninvasive solution, with risk mitigation for newborns. In the future, the device could be used, with basic instructions for caregivers at birthing locations, without previous expertise, as is needed to perform obstetric ultrasounds or neonatal maturity scores. The lack of birth care provided by skilled health personnel is a current concern occurring in approximately 30% the world and 50% in Africa (2007–2014) [21].

### Skin reflection and GA prediction

The analysis of the neonatal skin backscattering values, in response to different colors of LEDs, showed that the optical properties of the newborn’s skin can be studied at birth to aid in determining the maturity of the tissue, and secondarily calculate the gestational age. Infants that were born between 25 to 42 weeks of gestation presented increasing values of skin reflection to wavelengths of 470 nm and 630 nm. The optical device measured the backscattered light signal returned from the skin, using similar approaches to a previous report that estimated the skin fat thickness [18]. Multi-layer optical models of skin have been developed to study the effects of tissue structure on light propagation in order to determine skin thickness [22]. The skin optical properties are characterized by the absorption and scattering coefficient of the epidermis, dermis, and subcutaneous layers, according to their pigments, keratin, collagen, and blood distribution [23]. In the vascular dermis and subcutaneous layers, the main absorbers in the visible spectrum of light are hemoglobin, carotene, bilirubin, and water, while the scattering properties come from the fibrous structures, such as collagen [14]. We ascribed the lack of linear correlation between R_575_ and the GA to the hemoglobin absorption properties, based on the backscattering acquisitions from the skin.

**Fig 7:**
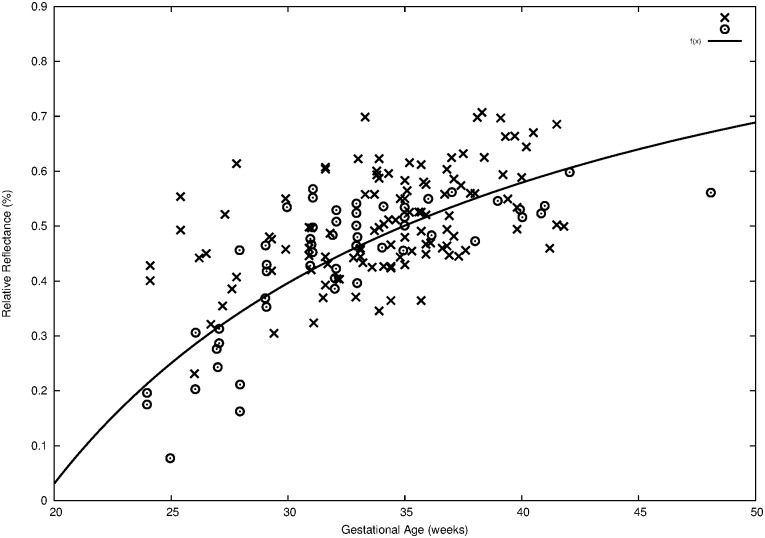
The skin reflectance versus gestational age comparing different studies. Cross symbol is the current reflectance data over the sole, at R_630_ 3.3; Dot symbol represents data by Lynn at al. [17]. The line is the correspondent reflectance equation fitted on our data: GA = 45.6 − 51.9*R_630_.

In a prior study, at 650 nm wavelength, the reduced-scattering coefficient increased linearly with gestational maturity for neonatal skin, deduced from the integrating-sphere measurements [24]. Similarly, our best correlations with the GA were at 630 nm. The R_630_ at 3.3 mm/6.5 mm ratio showed the importance of corrections for environment influences, such as ambient light and pressure of the sensor against the skin. At 470 nm and 630 nm, the skin reflectance response changed linearly with the GA, reproducing data from other reports. One of the studies, using the 450–750 nm range, demonstrated that the total reflectance of neonatal skin, shown by the reduced-scattering coefficients, increased linearly with gestational maturity, based on the integrating-sphere approach for measurements [24]. Another study calculated reflectance at 837 nm (R_837_), which was related exponentially to GA in 64 newborns at 24 to 42 weeks of gestation [17]. The authors showed that the dependence of collagen development affects the amount of optical scattering from the dermis. Despite the fact that the GA in that study was not based on the gold standard, we compared our results (cross symbol) to theirs (dot symbol), fitting our data from alive newborns together with the Lynn et al. data [17], as seen in Fig 7. The results indicated that the prototype used in our study yielded data comparable to the previous studies obtained with spectroscopes. We did not use the similar study of Post et al. to compare our data because the GA estimate was not as reliable as current methods, resulting in a gestation length of 44 weeks [16].

We need further analysis to understand the differences in the GA correlations between the forearm and the sole better. The neonatal skin is different from an adult in structure, function, and composition [25], mainly regarding the skin barrier function within the stratum corneum at the top of the epidermis [26]. The skin permeability barrier, formed during late gestation, is essential for neonatal survival. A report about the skin maturation in the human fetus demonstrated that this barrier formation coincides with the limit of the viability of pre-term infants 20 to 24 weeks [13], but extends until birth and through the first year of life [26]. Additionally, at the same gestational age, the skin of different places in the body, such as the abdomen and head, are in different stages of keratinization [13]. It could be an indication of new insights about the differences of optical properties between the skin of forearm and sole and non-explained outliers in our study. The skin colour and ethnicity are relevant variables to consider on the agenda for the future. It is still important to consider the topographical irregularity of the skin as a source of within-site or between-sites variations of their optical properties [27]. In the next version of our prototype, we will use synchronized and repetitive measurements at the same place to take the mean, instead of a single value, as a possible solution for the within-site variation.

### Statistical modeling choice

An equation including only the skin optical properties, R_630_ and R_470_ skin reflection together, explained 66.7% of the gold standard GA variability. Looking for an improved predictive model for GA, the skin reflection values were adjusted based on clinical variables.

In our sample, 78.3% of newborns were premature, 21.2% were small for gestational age, 14.8% were from twin gestation, and 7.8% had major malformations, reflecting actual settings of high complexity of care. This particular scenario is considered as part of the challenge in predicting the GA, in a health compromised population. We believe that clinical variables, easy to obtain at birthing locations, can improve the model based on the skin reflection.

Birth weight and the incubator staying variables improved the explanation regarding the GA variability, compiling weight values with R_630_ and R_470_ skin reflection. However, the relationship between birth weight and GA is known and this predictor alone is insufficient to estimate GA because size does not signify maturity [25]. The existence of both of the variables in the model could improve the equation, reinforcing their individual effects on the GA. Further study needs to be done on incubator staying to support or refute this initial evidence. In fact, humidity and temperature are environmental conditions associated with the optical properties of matter [28], and adjustments in the automatic calculations can be performed by the processor of the device.

Adjusted models are still important to evaluate confounding variables, such as the sex of the newborn and phototherapy, in the current analysis. The frequency of phototherapy during the first day of life was low in our studied group (10.4%). However, further studies are necessary to prove or disprove our initial results. Both size of a fetus based on ultrasound measurements, and sex, are recognized as contributing factors for standard growth [18]. This may be true for skin growth as well. The phototherapy influence on neonatal skin reflectance, as pointed out in a previous analysis [16] deserves further attention.

In terms of external validation, we expect to adjust the GA prediction model to include the challenges of identifying prematurity, in order to support health decisions, and this depends on addressing the complexity of prematurity. We understand that prematurity has a multifactorial etiology and this particular population has clinical complexity. Demographic, socioeconomic, medical, and health behavioral characteristics, as well as iatrogenic and spontaneous preterm birth, twinning are associated with prematurity, sometimes without a proper explanation [29]. Small size at birth is a risk factor associated with more than 80% of neonatal deaths and increases the risk of post-neonatal mortality [1]. A solution for predicting GA must address the inability of health systems to identify fetal growth failure, based on reliable information.

### Limitations and perspectives

A potential limitation of the present study is that reproducible measurement of CCI was possible in this first analysis, considering only two observers, accessing adult skin. Despite high coefficients of intraobserver and interobserver repeatability, three of twelve Bland–Altman plots deserve further investigation, mainly regarding the skin reflectance at 470 nm. The overall reproducibility requires confirmation in other centers and among more than two raters and preferably accessing premature and term newborns.

Parametric statistic tests are based on a theoretical probability normal distribution. The methodology was used to analyze the association between outcome and predictors, once the selected group of newborns resulted in a Gaussian distribution for the GA variable. However, in the actual birth setting, a nonparametric distribution of probability is expected for population-wide GA distribution because the prematurity rate is around 7.5% to 12.5% in the world, considering health inequities between countries [30]. Using this particular group in a cross-section design, the analysis proved the concept that newborn skin reflection is modified according to the skin growth, mirroring the gestational evolution. The main limitation of the models was generalizability. We prepared a statistical equation to explain the expected relationship between skin reflectance and GA that is not ready to be used in general population yet, but that is directed to the target population of premature infants.

Availability of reliable gestational age data is a prerequisite for preterm birth classification and health decisions [29]. Ultrasound machines and health professional training are costly, and they are not available or accessible enough to better predict GA in resource-constrained countries with fragile health care systems [6]. The skin reflection based on the new LED optical device value should be an affordable solution, but the approach needs a confirmatory study. A multiracial clinical prospective, cross-sectional multicenter study with reference standard and blinding, including low, medium, and high income countries, accepting small and large for gestational age newborns, is the next step. Additionally, artificial intelligence techniques can be utilized to improve the GA prediction.

## Supporting information

S1 Fig Intraobserver Bland–Altman plot for skin reflectance at 630 nm acquisition, at 3.3 mm distant of sensor. SD Standard deviation.
(EPS)

S2 Fig Intraobserver Bland–Altman plot for skin reflectance at 630 nm acquisition, at 6.5 mm distant of sensor. SD Standard deviation.
(EPS)

S3 Fig Intraobserver Bland–Altman plot for skin reflectance at 575 nm acquisition, at 4.0 mm distant of sensor. SD Standard deviation.
(EPS)

S4 Fig Intraobserver Bland–Altman plot for skin reflectance at 575 nm acquisition, at 7.2 mm distant of sensor. SD Standard deviation.
(EPS)

S5 Fig Intraobserver Bland–Altman plot for skin reflectance at 470 nm acquisition, at 3.3 mm distant of sensor. SD Standard deviation.
(EPS)

S6 Fig Intraobserver Bland–Altman plot for skin reflectance at 470 nm acquisition, at 6.5 mm distant of sensor. SD Standard deviation.
(EPS)

S7 Fig Interobserver Bland–Altman plot for skin reflectance at 630 nm acquisition, at 3.3 mm distant of sensor. SD Standard deviation.
(EPS)

S8 Fig Interobserver Bland–Altman plot for skin reflectance at 630 nm acquisition, at 6.5 mm distant of sensor. SD Standard deviation.
(EPS)

S9 Fig Interobserver Bland–Altman plot for skin reflectance at 575 nm acquisition, at 4.0 mm distant of sensor. SD Standard deviation.
(EPS)

S10 Fig Interobserver Bland–Altman plot for skin reflectance at 575 nm acquisition, at 7.2 mm distant of sensor. SD Standard deviation.
(EPS)

S11 Fig Interobserver Bland–Altman plot for skin reflectance at 470 nm acquisition, at 3.3 mm distant of sensor. SD Standard deviation.
(EPS)

S12 Fig Interobserver Bland–Altman plot for skin reflectance at 470 nm acquisition, at 6.5 mm distant of sensor. SD Standard deviation.
(EPS)

## Abbreviations

CCI: intraclass correlation coefficient
CI: confidence interval
GA: gestational age
LED: light emitting diode
LMP: last menstrual period
NICU: neonatal intensive care unit
R: skin reflectance (the measure of skin reflection)
SD: Standard deviation
